# Sniffing out cognitive decline in patients with and without evidence of dopaminergic deficit

**DOI:** 10.1016/j.prdoa.2019.09.002

**Published:** 2019-10-17

**Authors:** Francesca V. Lopez, Brittany Y. Rohl, Aparna Wagle Shukla, Dawn Bowers

**Affiliations:** a Department of Clinical and Health Psychology, University of Florida, Gainesville, FL, USA; b Department of Neurology and Fixel Center for Neurological Diseases, University of Florida, Gainesville, FL, USA

**Keywords:** Parkinson's disease, Dopaminergic deficit, Olfaction dysfunction, Cognitive decline, Longitudinal study

## Abstract

**Background:**

Depletion of dopamine is a major neuropathological feature of Parkinson's disease; however, 15% of patients with parkinsonian motor symptoms have neuroimaging evidence of intact dopaminergic function. Recent work has demonstrated that such patients without dopaminergic deficit are at a greater risk of cognitive impairment yet have intact olfaction relative to parkinsonian patients with dopaminergic deficit.

**Objectives:**

Given the high discriminatory power of olfaction assessments in movement disorders, the current study sought to determine whether olfaction dysfunction differentially predicted cognitive decline in patients with or without dopaminergic deficit.

**Methods:**

Data were obtained from the Parkinson's Progression Marker Initiative. The total sample included 401 patients with and 51 patients without dopaminergic deficit, based on neuroimaging scans, and 175 healthy controls. Participants were categorized into non-impaired or impaired olfaction groups based on performance on the University of Pennsylvania Smell Identification Test. Participants were administered the Montreal Cognitive Assessment twice (baseline and two-year follow-up), and change scores were calculated to examine changes in cognition over time.

**Results:**

Within the impaired olfaction groups, participants without dopaminergic deficit had lower cognitive scores than participants with dopaminergic deficit and healthy controls at baseline. Group differences were not significant at follow-up; rather, impaired baseline olfaction predicted cognitive decline across all study participants.

**Conclusions:**

Future studies are needed to assess whether the profile of motor and non-motor symptoms in patients without dopaminergic deficit, including olfaction, are deserving of their own syndrome, or whether individual patients may fit better under alternative, existing diagnoses.

## 1 Introduction

Dopamine transporter-single photon emission computer tomography (DaT-SPECT) is sensitive to presynaptic dopamine neuronal dysfunction, which is the major neuropathological feature of Parkinson's disease (PD; [1]). In large clinical trials, 5–15% of patients who are diagnosed with PD have normal DaT-SPECT imaging findings [2]. Patients with these imaging findings have been labeled as having scans without evidence of dopaminergic deficit (SWEDD). By definition, patients with SWEDD have parkinsonian motor symptoms yet have intact dopaminergic functioning. Because of this, whether SWEDD should be considered a subgroup of PD at all is controversial, and there are several competing hypotheses.

Initially, patients with SWEDD were thought to characterize patients in prodromal stages of PD; however, only 2–13% of these patients later received a formal PD diagnosis [3]. Alternatively, the motor symptom presentation in SWEDD may more closely align with adult-onset dystonia, essential tremor, or drug-induced parkinsonism [4]. For example, a recent meta-analysis reported that patients with SWEDD show clinical symptoms of dystonia with asymmetric resting tremor yet lack the clinical progression of motor symptoms and response to levodopa treatment seen in PD.

Prior work investigating the cognitive profile of patients with SWEDD has yielded mixed results. Initially, it was reported that newly diagnosed patients with SWEDD had lower scores on a global cognitive screener (i.e., Montreal Cognitive Assessment; MoCA; [5]) as compared to newly diagnosed patients with PD or healthy controls [6]. More recently, no differences were reported on more sensitive cognitive measures (i.e., visuospatial, working memory, recent memory, and executive function) between newly diagnosed patients with SWEDD and PD, with both showing similar occurrences (i.e., 25%) of mild cognitive impairment [7]. However, in relation to PD, newly diagnosed patients with SWEDD had greater decline on a global cognitive screener over a two-year period (i.e., MoCA; [8]). Notably, demographic characteristics (i.e., age and education) and disease duration did not differ between patients with SWEDD with and without cognitive decline. Consequently, the pattern of cognitive impairment and predictors of current or future cognitive functioning in patients with SWEDD remains unclear.

Olfaction disturbance is common in alpha-synucleinopathies (i.e., multiple system atrophy, dementia with Lewy Bodies, Parkinson disease) and other neurodegenerative diseases like Alzheimer's disease. Indeed, the prevalence of olfaction dysfunction is estimated to be between 80 and 90% in PD patients [9,10]. Although the underlying mechanisms are not well understood, olfaction disturbance often predates the manifestation of motor symptoms that are required for a clinical diagnosis of PD [11]. Conversely, prior studies have reported that patients with SWEDD have better olfaction performance as compared to patients with PD [12,13], and comparable performance to healthy controls [14]. Moreover, worse olfaction performance has been associated with a higher likelihood of a clinical PD diagnosis in patients with suspected PD [14]. Together, these findings echo the extant literature regarding the clinical value of olfactory assessment in distinguishing patients with PD from other neurological diseases that are often misdiagnosed as PD (i.e., essential tremor, dystonia, and vascular parkinsonism; [11]).

Olfaction disturbance is associated with non-normative cognitive decline in both PD and non-PD populations. Furthermore, patients with SWEDD may be at a greater risk of cognitive decline as compared to patients with PD. The question arises as to whether olfaction status (i.e., normal or impaired) is associated with current or future cognitive decline in patients with SWEDD. Thus, the overall goal of the current study was to examine the longitudinal relationship between olfaction performance and changes in cognition over a two-year period in newly diagnosed patients with PD or SWEDD relative to healthy older adults. There were two aims of this study. Aim 1 examined the relationship between baseline olfaction and cognition in patients with PD or SWEDD. Based on prior findings, we predicted that patients with impaired olfaction, regardless of PD or SWEDD classification, would have worse cognitive performance relative to healthy controls. Aim 2 examined whether baseline olfaction differentially predicted changes in cognition over time in patients with SWEDD relative to those with PD or healthy controls. We predicted that baseline olfactory impairment would be associated with greater declines in cognitive performance over a two-year period in patients with SWEDD relative to patients with PD or healthy controls.

## 2 Method

### 2.1 Data acquisition

Archival data were obtained from the Parkinson's Progression Marker Initiative (PPMI) database. For information about the aims and methodology of the PPMI study, see Marek et al. [15] and the PPMI website (www.ppmi-info.org). The PPMI research protocol and data collection methods were approved by the institutional review boards at each participating PPMI data collection site. The PPMI obtained written, informed consent from all study participants before they enrolled.

### 2.2 Participants

Participants (*n* = 627) consisted of individuals with idiopathic PD (*n* = 401), SWEDD (*n* = 51), and healthy controls (HC; *n* = 175). All individuals in the PD or SWEDD group had been diagnosed with new onset idiopathic PD within the previous twenty-four months and none were taking PD medications at the time of PPMI enrollment. Determination of PD or SWEDD group membership was based on DaT-SPECT imaging findings (i.e., the presence or absence of dopamine deficiency). For information about the DaT-SPECT image processing protocols and procedures for calculation of striatal binding ratios, see PPMI website (www.ppmi-info.org). Exclusion criteria for the HC group included abnormal imaging findings (e.g., MRI, DaT-SPECT), history of neurologic disease, motor symptoms, first degree relative with PD, or cognitive impairment as defined by a cutoff score of ≤26 on the Montreal Cognitive Assessment (MoCA; [5]). All participants were screened for depressive symptoms using the Geriatric Depression Scale-15 (GDS-15; [16,17]), a brief 15-item “yes-no” scale, with higher scores reflecting greater depressive symptoms. Disease severity was assessed using the Modified Hoehn and Yahr staging scale (H&Y; [18]). The scale ranges from 0 (“No visible symptoms of PD”) to 5 (“PD symptoms on both sides of the body and unable to walk”), with higher scores reflecting more advanced disease stage. Motor severity was assessed using the motor scale from the Movement Disorder Society-Unified Parkinson's disease rating scale (MDS-UPDRS; [19]) Part III, where Part III total scores range from 0 to 108; higher scores reflect a greater amount of and/or more severe motor symptoms. See Table 1 for demographic information of study participants.

**Table 1:** Demographic characteristics of the sample (*n* = 627).

	PD group (*n* = 401)	SWEDD group (*n* = 51)	HC group (*n* = 175)	Test statistic
Demographics				
Age (years)	65.0 (10.3)	65.9 (10.6)	67.5 (11.1)	*F*(2, 624) = 3.37^a^
Education (years)†	15.6 (2.99)	15.6 (3.03)	16.1 (2.97)	*χ*^*2*^(2, N = 627) = 7.039
Gender (M/F)	137/291	19/46	61/138	*χ*^*2*^(2, N = 627) = 0.27
Handedness (R%)	88.3	80.0	82.4	*χ*^*2*^(4, N = 627) = 4.19
Disease duration (months)	8.21 (7.42)	8.17 (7.82)	–	*F*(1, 450) = 0.138
Baseline				
UPDRS Part III total†	20.7 (9.11)	14.9 (11.6)	1.26 (2.27)	*χ*^*2*^(2, N = 627) = 376^b^
Modified Hoehn and Yahr Stage (%)				
Stage 0	0.2	–	98.9	
Stage 1	44.4	54.9	1.1	
Stage 2	54.9	45.1	–	
Stage 3	0.5	–	–	
GDS-15 total†	2.32 (2.47)	3.49 (3.83)	1.31 (2.15)	*χ*^*2*^(2, N = 627) = 43.6^c^
Follow up				
UPDRS Part III total†	23.2 (11.2)	16.2 (13.7)	1.51 (3.02)	*χ*^*2*^(2, N = 627) = 362^b^
Modified Hoehn and Yahr Stage (%)				
Stage 0	0.3	23.5	98.3	
Stage 1	27.8	41.2	1.1	
Stage 2	67.6	29.4	0.6	
Stage 3	3.5	5.9	–	
Stage 4	0.8	–	–	
GDS-15 total†	2.62 (2.89)	3.16 (3.15)	1.19 (1.94)	*χ*^*2*^(2, *N* = 578) = 53.9^c^

Notes: UPDRS = Unified Parkinson's disease Rating Scale; GDS = Geriatric Depression Scale.

a Bonferroni post hoc for the following between group comparisons at *p* < .05: PD < HC

† Kruskal Wallis' with post-hoc Wilcoxon-Mann-Whitney's *U* tests corrected for multiple comparisons setting the *p* values at *p* < .01:

b PD > SWEDD > HC

c PD = SWEDD > HC

### 2.3 Materials and procedure

Baseline olfaction was assessed using the University of Pennsylvania Smell Identification Test (UPSIT; [20]). The UPSIT consists of four enveloped sized booklets, each containing 10 forced-multiple choice “scratch and sniff” items. As outlined in the PPMI protocol (see Marek et al. [15]), Hyposmic (e.g., impaired olfaction) group membership was determined as <10th percentile, using UPSIT normative values for age and gender. Global cognitive functioning was assessed using the Montreal Cognitive Assessment (MoCA; [5]), corrected for education. Total scores from 0 to 30, with higher scores reflecting better global cognitive performance. See Table 2 for baseline UPSIT group membership and MoCA performance of the study participants.

**Table 2:** Olfaction group membership and cognitive performance of the sample (n = 627).

	PD group (*n* = 401)	SWEDD group (*n* = 51)	HC group (*n* = 175)
Baseline UPSIT group (n)			
Normosmia	37 (9.2%)	23 (45.1%)	111 (63.4%)
Hyposmia	364 (90.8%)	28 (54.9%)	64 (36.6%)
Cognitive screener			
Baseline MoCA total	27.1 (2.32)	27.0 (2.41)	28.2 (1.09)
MoCA change score	−0.93 (2.69)	−1.29 (3.11)	−1.06 (2.34)

Notes: UPSIT = University of Pennsylvania Smell Identification Test; MoCA = Montreal Cognitive Assessment. Raw means and standard deviations are presented.

### 2.4 Statistical analyses

MoCA Change Scores were calculated by subtracting MoCA raw scores at baseline from raw scores at follow up. Negative change scores denote declines in global cognitive functioning (see Table 2). Chi-square tests were performed to compare all three groups in UPSIT group membership, gender, and handedness. One-way analyses of variances (ANOVAs) were performed to compare groups on age and MoCA Change Scores. Owing to non-normality of the data, Kruskal Wallis H-Tests were performed to compare all three groups on the following variables: education, MDS-UPDRS Part III total scores (baseline and follow up), GDS-15 total scores (baseline and follow up), and MoCA total scores. Corrections for multiple comparisons were imposed. Owing to non-normality of the data, two separate bias-corrected bootstrapped ANOVAs using 1000 samples were performed on baseline MoCA total scores or MoCA Change Scores. Significant interactions were followed-up with bias-corrected bootstrapped pairwise comparisons.

## 3 Results

### 3.1 Prevalence

Table 2 shows the distribution of individuals in the Hyposmic and Normosmic groups according to clinical diagnosis (i.e., PD, SWEDD, or HC). Overall, the percentage of participants assigned to each UPSIT group significantly differed, [χ^2^ (2, *N* = 627) = 189, *p* < .001]. In the PD group, 90.8% of participants met criteria for the Hyposmic group (i.e., PD+) and the remaining met criteria for the Normosmic group (i.e., PD−). This represents a significant difference [χ^2^ (1, *N* = 401) = 16.3, *p* < .001]. In the SWEDD group, there was a more equal distribution of individuals in the Hyposmic (53.8%, SWEDD+) and Normosmic groups (46.2%, SWEDD−), which is not significantly different, [χ^2^ (1, *N* = 51) = 0.560, *p* = .575]. Finally, more individuals in the HC group (63.4%; HC−) met criteria for being included in the Normosmic group than the Hyposmic group (36.6%; HC+). This difference is significant, [χ^2^ (1, *N* = 175) = 3.47, *p* = .001].

### 3.2 Disease stage of SWEDD group

Based on the observed pattern of change in H&Y staging of the SWEDD group from Baseline to 2-year Follow-up (see Table 1), a question was raised as to whether the change was associated with worse olfaction or cognition. The majority of participants in SWEDD group remained in the same or reverted back to an earlier disease stage. Of the six remaining participants in the SWEDD group, change in H&Y was unrelated to baseline olfaction status [*r*_Spearman's rho_ = 0.189, *p* = .132] or current [*F*(1, 50) = 1.06, *p* = .306] and changes [*F*(1, 50) = 2.09, *p* = .155] in global cognitive functioning.

### 3.3 Baseline MoCA performance

To examine the relationship between baseline olfaction and cognitive performance, a 3 (Diagnosis Group: HC vs. PD vs. SWEDD) × 2 (UPSIT Group: Normosmic vs. Hyposmic) ANOVA was performed on baseline MoCA total scores. There was a significant main effect of Diagnosis Group, [*F*(2, 621) = 10.2, *p* < .001]. Bootstrapped Bonferroni pairwise comparisons revealed that, on average, the HC group (*M* = 28.2, *SD* = 1.09) had significantly higher baseline MoCA total scores than the PD (*M* = 27.1, *SD* = 2.32) or SWEDD (*M* = 27.0, *SD* = 2.41) groups, all *p*s = 0.001. The PD and SWEDD groups had comparable performance, *p* = .561. There was a significant main effect of UPSIT Group, [*F*(1, 621) = 13.2, *p* < .001]. Bootstrapped Bonferroni pairwise comparisons revealed that, on average, the Hyposmic group (*M* = 27.2, *SD* = 2.20) had significantly lower MoCA baseline total scores than the Normosmic group (*M* = 28.0, *SD* = 1.72), *p* = .003.

**Fig. 1:**
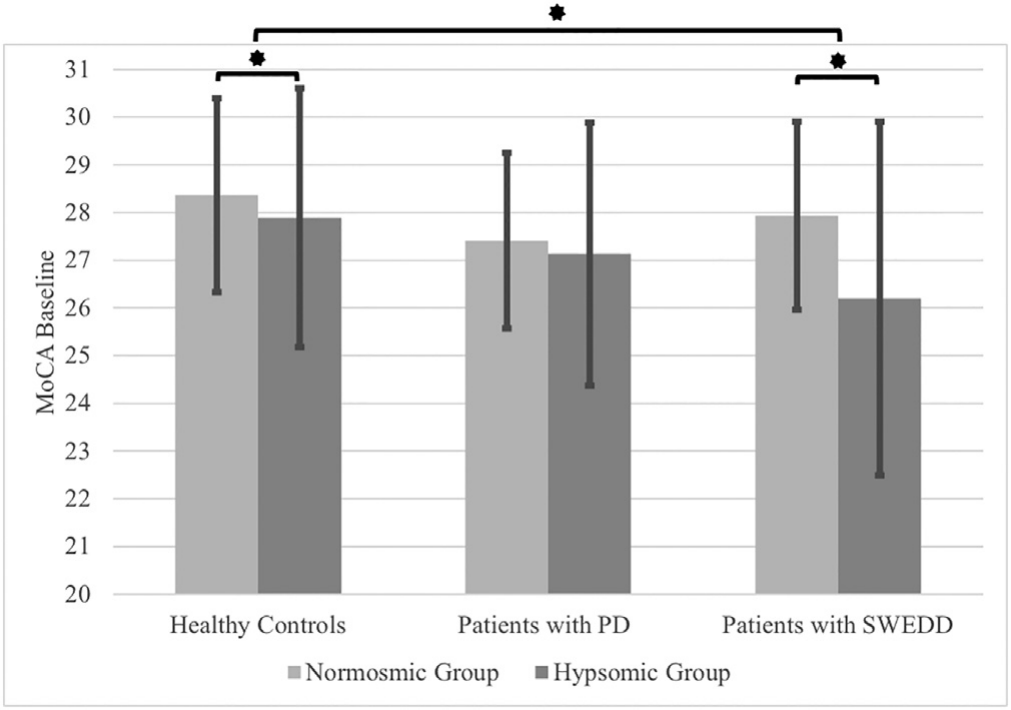
Baseline MoCA Performance of Patients with SWEDD and PD Varied as a Function of Baseline UPSIT Group Membership (Normosmic vs. Hyposmic Group). *Notes*: Summary t-test between HC and SWEDD groups, [*t*(79.0) = 25.6, *p* < .001]. Samples sizes across groups (total n = 627) n_HC-_ = 111; n_HC+_ = 64; n_PD-_ = 37; n_PD+_ = 364; n_SWEDD-_ = 23; n_SWEDD+_ = 28. Error bars represent standard deviations. *p < .01

As shown in Fig. 1, these main effects were qualified by a significant Diagnosis Group × UPSIT Group interaction, [*F*(2, 621) = 3.01, *p* = .05]. For the Hyposmic groups, bootstrapped pairwise Bonferroni comparisons indicated that the HC+ group (*M* = 28.0, *SD* = 1.07) had significantly higher baseline MoCA total scores than both the PD+ (*M* = 27.1, *SD* = 2.79) and SWEDD+ groups (*M* = 26.4, *SD* = 2.75) groups, all *p*s < 0.01. Further, the baseline MoCA of the PD+ group was significantly higher than that of the SWEDD+ group, *p* = .05. For the Normosmic groups, bootstrapped Bonferroni pairwise comparisons indicated that the HC− group (*M* = 28.3, *SD* = 1.08) had significantly higher baseline MoCA total scores than the PD− group (*M* = 27.3, *SD* = 2.79, *p* = .029). The SWEDD− group (*M* = 27.8, *SD* = 1.68) had comparable performance to both the HC− and PD− groups, all *p*s > 0.183.

### 3.4 MoCA change scores

**Fig. 2:**
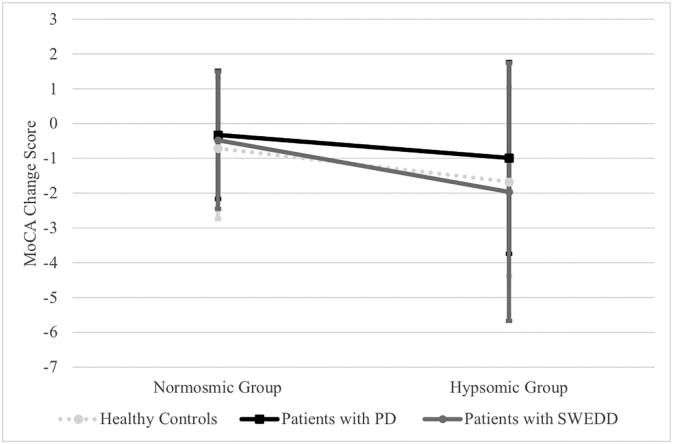
Baseline UPSIT Group Membership (Normosmic vs. Hyposmic Group) Predicts Change in MoCA Performance of Study Participants. *Notes:* Sample sizes are as follows (total n = 627) n_HC-_ = 111; n_HC+_ = 64; n_PD-_ = 37; n_PD+_ = 364; n_SWEDD-_ = 23; n_SWEDD+_ = 28. Error bars represent standard deviations.

To determine whether baseline olfaction impairment was associated with changes in cognitive performance, a 3 (Diagnosis Group: HC vs. PD vs. SWEDD) × 2 (UPSIT Group: Normosmic vs. Hyposmic) ANOVA was performed on MoCA Change Scores. There was a significant main effect of UPSIT Group, [*F*(2, 621) = 10.6, *p* = .001]. Bootstrapped pairwise Bonferroni comparisons revealed that, on average, the Hyposmic Group (*M* = −1.54, *SD* = 2.82) demonstrated a significance one-point decline compared to the Normosmic Group (*M* = −0.505, *SD* = 1.98) on the MoCA over a two-year period, *p* = .003. The main effect of Diagnosis Group (*F*(2, 621) = 1.78, *p* = .170) and the Diagnosis Group × UPSIT Group interaction (*F*(2, 621) = 0.463, *p* = .629) were non-significant. See Fig. 2.

## 4 Discussion

The present study is the first study, to our knowledge, to directly compare cognitive performance between PD or SWEDD patients with and without olfaction disturbance. We found that patients with hyposmia, either PD+ or SWEDD+, had worse baseline cognitive performance than healthy controls with hyposmia (HCs+). Further, patients with SWEDD+ had worse cognitive performance as compared to PD+. While previous work examining cognitive status in patients with SWEDD has been mixed [6–8], our findings demonstrate the clinical utility of olfaction assessments. Without taking olfaction status into consideration, results would have suggested that patients with PD or SWEDD have comparable cognitive baseline scores, which were significantly lower than healthy controls. However, when taking olfaction status into consideration, results indicated that patients with SWEDD+ had significantly worse baseline cognitive performance relative to both PD+ and HC+ groups. With that said, performance of SWEDD+ participants, on average, was within normal limits (i.e., ≥26). Given the regularity of olfaction disturbance in non-PD and PD populations coupled with our finding that SWEDD patients, on average, had the tendency to demonstrate greater declines on the MoCA than PD patients, the presence of olfaction dysfunction may likely reflect risk for pathological cognitive changes rather than suggestive of a clinical diagnosis of PD. However, the degree to which these changes are related to the “true” diagnosis of patients with SWEDD remains unclear. Together, these findings may imply that olfaction screeners may have the potential to provide insight into current cognitive functioning in patients with SWEDD.

We failed to find that baseline olfaction dysfunction differentially predicted cognitive decline, on the MoCA, in patients with SWEDD+ relative to patients with PD+ or HCs+. Rather, baseline olfaction disturbance was associated with a *reliable* MoCA decline (i.e., difference = 1.5, Reliable Change Index = 2.83), regardless of group (PD, SWEDD, HC) over a two-year period. Although the interaction failed to reach significance, the difference in means between patients with SWEDD+ or PD+ was associated with a moderate effect size (i.e., Cohen's d = 0.55), suggesting that patients with SWEDD+ may be at a greater risk of future cognitive decline than patients with PD+, independent of motor severity. One prior study using the MoCA reported that patients with SWEDD were at a greater risk of cognitive decline over a two-year follow-up compared to patients with PD [8]. That study differed from ours in methodology; however, in that the investigators examined solely dopaminergic deficit group differences on MoCA total scores, and they examined follow-up scores on the MoCA rather than decline (follow-up - baseline). Since changes in cognitive performance were assessed with a global cognitive screener (i.e., MoCA), future research should investigate the cognitive domains most susceptible to decline in patients with PD or SWEDD using a comprehensive neuropsychological battery and examine the extent to whether these relationships with olfaction disturbance are causal. Furthermore, Wyman-Chick et al. [8] as well as the current study utilized data from the same dataset (i.e., PPMI), which highlights the importance of investigating the relationship between olfaction and cognitive performance in individuals with and without dopaminergic deficits using an external dataset. Such an approach may assist in differentiating alternative disease processes that may help to explain normal DaT-SPECT imaging findings in SWEDD.

In a broader context, these results parallel previous findings between olfactory function and cognitive performance in both PD and non-PD populations [[21], [22], [23]]. Specifically, hyposomnia is a common feature seen in Alzheimer's [24] and Parkinson's disease [25] as well as dementia with Lewy Bodies [26]. Moreover, these olfaction disturbances have been hypothesized to stem from disruption of cholinergic systems which subsequently have deleterious effects on cognitive functioning in these clinical populations [[24], [25], [26]]. Thus, these findings provide further evidence that the relationship between olfactory dysfunction and cognitive performance may generalize to multiple clinical populations, including patients with SWEDD. Nonetheless, odor identification is a single indicator of olfactory function, and the degree to which other indicators of olfactory function (e.g., odor discrimination, memory, intensity, and pleasantness) are impaired in patients with SWEDD is unknown. Subsequent investigators should consider examining olfaction across the spectrum of function to determine whether the profile olfaction impairments are similar between patients with SWEDD or PD.

There are a few notable limitations of the current study. Most of the participants were highly educated and primarily White which may limit the generalizability of the results to less-educated and non-majority populations. In addition, information regarding health conditions known to affect olfactory function (i.e., history of sinus infection, tobacco use, or insult) was not available for participants in the current study [27]. Lastly, depressive symptoms were unrelated to global cognitive and olfaction performance in the current study; however, it remains to be determined whether symptoms of anxiety or apathy contribute to this relationship in patients with SWEDD or PD.

Overall, the current study suggests that clinicians may wish to monitor olfaction in patients with SWEDD. Specifically, our findings suggest that global cognitive screeners are particularly useful for identifying individuals with current cognitive dysfunction as well as those at-risk of future cognitive decline when used in conjunction with an olfaction screener. Furthermore, the findings of the current study warrant subsequent investigation to determine whether olfaction assessment has diagnostic merit in patients with SWEDD. Since the progression of patients with SWEDD may involve complex interactions among a variety of motor and non-motor symptoms, future studies are needed to determine the interaction among multiple risk and protective factors related to changes in cognition in patients with SWEDD.
